# Zinc Regulates Glucose Metabolism of the Spinal Cord and Neurons and Promotes Functional Recovery after Spinal Cord Injury through the AMPK Signaling Pathway

**DOI:** 10.1155/2021/4331625

**Published:** 2021-07-31

**Authors:** Hengshuo Hu, Nan Xia, Jiaquan Lin, Daoyong Li, Chuanjie Zhang, Minghao Ge, He Tian, Xifan Mei

**Affiliations:** ^1^Department of Orthopedics, The First Affiliated Hospital of Jinzhou Medical University, Jinzhou, Liaoning, China; ^2^Pharmacy School, Jinzhou Medical University, Jinzhou, Liaoning, China; ^3^Graduate Training Base of Jinzhou Medical University, Chaoyang Central Hospital, Chaoyang, Liaoning, China; ^4^Department of Histology and Embryology, Jinzhou Medical University, Jinzhou, Liaoning, China

## Abstract

Spinal cord injury (SCI) is a traumatic disease that can cause severe nervous system dysfunction. SCI often causes spinal cord mitochondrial dysfunction and produces glucose metabolism disorders, which affect neuronal survival. Zinc is an essential trace element in the human body and plays multiple roles in the nervous system. This experiment is intended to evaluate whether zinc can regulate the spinal cord and neuronal glucose metabolism and promote motor functional recovery after SCI. Then we explore its molecular mechanism. We evaluated the function of zinc from the aspects of glucose uptake and the protection of the mitochondria in vivo and in vitro. The results showed that zinc elevated the expression level of GLUT4 and promoted glucose uptake. Zinc enhanced the expression of proteins such as PGC-1*α* and NRF2, reduced oxidative stress, and promoted mitochondrial production. In addition, zinc decreased neuronal apoptosis and promoted the recovery of motor function in SCI mice. After administration of AMPK inhibitor, the therapeutic effect of zinc was reversed. Therefore, we concluded that zinc regulated the glucose metabolism of the spinal cord and neurons and promoted functional recovery after SCI through the AMPK pathway, which is expected to become a potential treatment strategy for SCI.

## 1. Introduction

Spinal cord injury (SCI) is a critical central nervous system disease that can cause permanent damage to nerve function, which is considered to be the main cause of paralysis [1]. Around 300,000 people worldwide suffer from SCI every year, which brings devastating blows to individuals and families and creates a heavy economic burden on society [2]. SCI is divided into primary injury and secondary injury. Primary injury caused by trauma leads to a series of complex secondary injuries, including oxidative stress, neuronal apoptosis, inflammation, mitochondrial dysfunction, and metabolic disorders [3, 4]. Neurons are particularly sensitive to changes in energy after SCI. Insufficient energy supply caused by impaired ATP synthesis seriously affects the survival of the neurons [5]. Metabolism changes are closely related to the survival of the neurons, but there are currently few studies on the spinal cord and neuronal metabolism after SCI, especially glucose metabolism, which is a problem worthy of attention.

Glucose is the main energy source for the central nervous system [6]. Ischemia and hypoxia after SCI lead to insufficient glucose supply, which is not conducive to the repair of the neurons [4]. The uptake of glucose depends on glucose transporters, and the main glucose transporters expressed in the neurons are GLUT3 and GLUT4 [7]. Damage can lead to changes in the function of glucose transporters, which in turn lead to insufficient glucose uptake and metabolic disorders [8]. As we all know, the principal place of glucose metabolism is the mitochondria. Mitochondrial damage after SCI is an important aspect of secondary injuries, which seriously affects the utilization of glucose. Decreased glucose utilization inhibits neuronal activity. Mitochondrial dysfunction observed in many diseases can cause the respiratory chain to break, reduce production capacity of energy, and promote neuronal death [9]. Oxidative stress is a highly disordered metabolic process, and ischemia and hypoxia after SCI can result in a sharp increase of reactive oxygen species (ROS) [1]. Mitochondria are the main targets of ROS detrimental effects, and ROS is the main cause of mitochondrial dysfunction [10]. Therefore, inhibiting oxidative stress is a key step to protect the function of the mitochondria and is essential for glucose metabolism. AMP-activated protein kinase (AMPK), a serine/threonine protein kinase, participating in numerous biological functions, is a key factor in glucose metabolism and is closely related to glucose transport and mitochondrial function [11, 12].

Zinc is part of the essential trace elements for biological development [13]. It combines with many enzymes and transcription factors, and participates in many biological functions such as immune response, cell homeostasis, cell apoptosis, and oxidative stress [14, 15]. Studies have indicated that zinc also played a central role in the nervous system, and zinc deficiency could lead to neuronal death [16]. Our previous studies have indicated that zinc had a protective effect on the neurons, reduced oxidative stress, and was beneficial to the recovery of SCI [17, 18]. However, the role of zinc in spinal cord and neuronal glucose metabolism after SCI is not yet clear. In this study, we explored the regulatory effects of zinc on the glucose metabolism of the neurons and spinal cord after SCI from two aspects of glucose uptake and mitochondrial protection, and clarified the role of AMPK in it, providing a new idea for the treatment of SCI.

## 2. Materials and Methods

### 2.1. Cell Culture and Treatment

In order to comprehend the biological effects of zinc on the neurons, PC12 cells were selected. PC12 cells were cultured in DMEM medium (Gibco, USA) replenished with 1% penicillin-streptomycin and 10% FBS in a humidified incubator at 37°C with 5% CO_2_. The cells were divided into four treatment groups: control group (cells were treated with 80 *μ*M glucose for 24 hours), H_2_O_2_ group (cells were treated with 80 *μ*M glucose for 24 hours after stimulated with 60 *μ*M H_2_O_2_ (Sigma-Aldrich) for 3 hours), H_2_O_2_+zinc group (cells were treated with 80 *μ*M zinc gluconate (Biotopped, Beijing, China) for 24 hours after stimulated with 60 *μ*M H_2_O_2_ for 3 hours), and H_2_O_2_+zinc+compound C group (cells were treated with 80 *μ*M zinc gluconate and 10 *μ*M compound C (Sigma-Aldrich) for 24 hours after stimulated with 60 *μ*M H_2_O_2_ for 3 hours) [19]. All drugs were diluted with complete culture medium.

### 2.2. Cell Viability Assay

The MTT assay was used to detect cell viability. Cells were seeded in 96-well plates, in a CO_2_ incubator at 37°C overnight, at a density of 5000 cells/well. Cells were treated with serial range of concentrations (0, 10, 20, 40, 50, 60, 100, 200, and 300 *μ*mol/l) H_2_O_2_ for 1, 3, 12, and 24 h or (0, 10, 20, 40, 60, 80, 100, 150, 200, and 500 *μ*mol/l) zinc gluconate for 24 h, with 100 *μ*l solution in each well. Then, 20 *μ*l of MTT solution (5 mg/ml in PBS) was added to each well for 4 h in the incubator. The MTT solution was removed, and MTT crystals were dissolved by 150 *μ*l DMSO. Finally, the optical density (OD) was read at 490 nm using a microplate reader.

### 2.3. Apoptosis Assay

Apoptosis was detected using the Mitochondrial Membrane Potential and Apoptosis Detection Kit (Beyotime, China) according to the manufacturer's instructions. Cells retaining their mitochondrial membrane potential were marked with red fluorescence (MitoTracker Red CMXRos), while the cells undergoing apoptosis were marked with green fluorescence (Annexin V-FITC). The kit also provided a Hoechst 33342 staining solution for fluorescent staining of the nucleus.

The apoptosis after zinc gluconate treatment was further investigated using Annexin V/PI apoptosis assay kit by flow cytometry [20].

### 2.4. Glucose Uptake Assay

Utilizing 2-NBDG (Invitrogen, Carlsbad, CA, USA), glucose uptake assay was performed as described previously [21]. 2-NBDG solution was diluted to 100 *μ*mol/l with KRH buffer. Cells were seeded in confocal plates. After treatment with drugs, PC12 cells were treated with 2-NBDG diluted solution for 30 minutes at 37°C. The cells were noted with a high-resolution confocal microscope. The fluorescence intensity was analyzed by ImageJ.

### 2.5. Immunofluorescence Analysis

Cells were seeded in a 24-well plate and processed accordingly. All cells were washed 5 times with PBS for 3 minutes and fixed in 4% PFA for 20 minutes. After washed by PBS, cells were incubated for 15 minutes in 0.1% Triton X-100 and then cultured for 2 h with 5% goat serum. Then, cells were incubated with the primary antibody at 4°C overnight. Next, the cells were incubated with secondary antibody for 2 hours at room temperature. Nuclei were stained with 4′,6-diamidino-2-phenylindole (DAPI) (Invitrogen, USA) solution for 15 min. The cells were then imaged with a fluorescent microscope (Olympus, Tokyo, Japan), and the results were dealt with by ImageJ. The following antibodies were used: anti-GLUT4 (1 : 200, Affinity, USA), anti-GLUT3 (1 : 200, Affinity, USA), anti-HO-1 (1 : 200, Affinity, USA), anti-NQO1 (1 : 200, Affinity, USA), anti-NRF2 (1 : 200, Affinity, USA), anti-NeuN (1 : 1000, Abcam), TRITC phalloidin (1 : 200, Solarbio, Beijing, China), and Alexa Fluor 488/568 goat anti-mouse IgG and Alexa Fluor 568 goat anti-rabbit IgG (1 : 1000, Thermo Fisher Scientific).

### 2.6. Detection of Intracellular ROS

Cells were seeded in a 24-well plate and processed accordingly. Intracellular ROS was detected by ROS Assay Kit (Solarbio, Beijing, China). Cells were cultured with DCFH-DA at 37°C for 20 min then observed with a fluorescent microscope.

### 2.7. Measurement of Mitochondrial Membrane Potential

Cells were seeded in confocal plates and treated accordingly. Mitochondrial membrane potential (MMP) was evaluated by MMP Assay Kit with JC-1 (Solarbio, Beijing, China) according to the manufacturer's instructions. When the MMP is high, JC-1 can produce red fluorescence. When it is weak, JC-1 generates green fluorescence. It is quite convenient to detect the change of MMP by the change of fluorescence color. The relative ratio of red to green fluorescence is often used to assess mitochondrial depolarization. All images were obtained with a confocal microscope.

### 2.8. Animal Model and Treatment

SPF-grade C57BL/6J mice (the same number of males and females, weighing 20-25 g, 8 weeks old) for this study were purchased from Liaoning Changsheng Biotechnology Company Ltd. (Benxi, China). These animals were maintained in a suitable environment with a 12 h light/dark cycle at 22 ± 2°C. Mice had free access to food and water and were housed for one week before the experimental performance. All of the experiment's programs used in this study were endorsed by the Animal Protection and Use Committee of Jinzhou Medical University.

All animals were intraperitoneal anesthesia with 50 mg/kg pentobarbital sodium, and the model of SCI was established by the weight-drop method as we previously described [22]. Briefly, a laminectomy was performed at the T9-T10 level and moderate contusion injury was made by dropping an impactor (2 mm diameter, 12.5 g, 2 cm in height) onto the surface of the spinal cord. Two hours after surgery, mice were treated with zinc gluconate (30 mg/kg, intraperitoneally) with and without compound C (10 mg/kg, intraperitoneally) [23]. Postoperative care was given, and the mice were given the drug daily until the third day. Sham group mice received a laminectomy except contusion and were injected with an equivalent dose of isosmotic glucose.

The mice were randomly divided into four groups: sham group (treated with isosmotic glucose), SCI group (treated with isosmotic glucose), SCI+zinc group (treated with zinc gluconate), and SCI+zinc+compound C group (treated with zinc gluconate and compound C).

### 2.9. In Vivo Imaging with PET

At 3 days post injury, glucose uptake was established by PET imaging of the biodistributions of ^18^F-FDG [24, 25]. The night before the experiment, mice were placed with no food but free access to water. The mice were anesthetized with isoflurane and injected with 13 *μ*Ci/g ^18^F-FDG through the tail vein. Forty minutes after treatment injection, mice were performed PET scan for 20 minutes. After data reconstruction, a total of 19.5 mm^2^ regions of 8 mm above and below the spinal cord injury site were selected as the region of interest (ROI). Calculating and comparing the average standardized uptake value (SUV) of each ROI.

### 2.10. TEM

Transmission electron microscopy (TEM) was used to monitor mitochondrial injury in the spinal cord, as described previously [26]. The structure of the mitochondria of the spinal cord in different groups of mice was observed, and the vacuole rate was calculated.

### 2.11. Western Blotting (WB) Analysis

Spinal cord tissues or cells were collected for WB assay as we described previously [22]. The following antibodies were used: anti-cleaved caspase 3 (1 : 1000, Affinity, USA), anti-Bax (1 : 1000, Affinity, USA), anti-Bcl-2 (1 : 1000, Affinity, USA), anti-PGC-*α* (1 : 1000, CST, USA), anti-NRF2 (1 : 1000, CST, USA), anti-p-AMPK (1 : 1000, CST, USA), anti-AMPK (1 : 1000, CST, USA), anti-GLUT4 (1 : 1000, Affinity, USA), anti-GLUT3 (1 : 1000, Affinity, USA), anti-HO-1 (1 : 1000, Affinity, USA), anti-NQO1 (1 : 1000, Affinity, USA), anti-*β*-actin (1 : 1000, Proteintech, USA), HRP-conjugated AffiniPure Goat Anti-Mouse IgG (1 : 10000, Proteintech, USA), and HRP-conjugated AffiniPure Goat Anti-Rabbit IgG (1 : 10000, Proteintech, USA).

### 2.12. Behavioral Assessment

Behavioral assessment was assessed using the Basso Mouse Scale (BMS) score [27]. At 1, 3, 5, 7, 10, 14, and 28 days after the operation, the mice were evaluated by two trained examiners in a double-blind manner. In BMS score ranging from 0 to 9, 0 indicated no ankle movement and 9 indicated complete normality.

At 28 days after injury, footprint analysis was performed [28]. The mice were coated with different dyes on their front and rear limbs, placed on absorbent paper surrounded by wooden boards, and requested to walk in a straight line (front limbs were coated with black dye and rear limbs were coated with red dye). Stride length and stride width were counted.

### 2.13. Histological Assessment

At 28 days after the operation, animals were anesthetized and then intracardially perfused with 0.9% normal saline followed by 4% PFA. 1 cm of spinal cord tissue centered on the injury point was removed and then fixed, dehydrated, embedded, and sliced. HE and Nissl staining were performed by Hematoxylin-Eosin Staining Kit (Solarbio, Beijing, China) and Nissl Staining Solution (Methylene Blue) (Solarbio, Beijing, China) according to the manufacturer's instructions. All images were captured under an optical microscope (DMI4000B, Leica, Wetzlar, Germany). For Nissl staining, we counted the number of ventral motor neuron (VMN) in spinal anterior horn of sections as described previously [29].

### 2.14. TUNEL Staining

TUNEL staining was performed with sections taken 3 days after injury by One Step TUNEL Apoptosis Assay Kit (Beyotime, China). In short, washed by PBS five times for three minutes, sections were incubated at room temperature with 0.5% Triton X-100 for five minutes. After PBS washing, 50 *μ*l TUNEL solutions was added into each sample at 37°C for 60 minutes in darkness. Washed by PBS three times, the sections were sealed with an antifluorescence quenching solution and then observed under a fluorescence microscope. Green fluorescence represented apoptosis cells.

### 2.15. Statistical Analysis

SPSS Software (version 25.0, Chicago, IL, USA) was used to perform statistical analysis. All data are presented as the mean ± standard deviation (SD). One-way analysis of variance (ANOVA) followed by Bonferroni post hoc test was used to compare multiple groups. BMS scores were analyzed by two-way ANOVA followed by Tukey's post hoc test. When the variance was not equal, the Kruskal-Wallis test was used. *p* < 0.05 was considered to be statistically significant.

## 3. Results

### 3.1. Zinc Inhibited Apoptosis of PC12 Neurons

**Figure 1:**
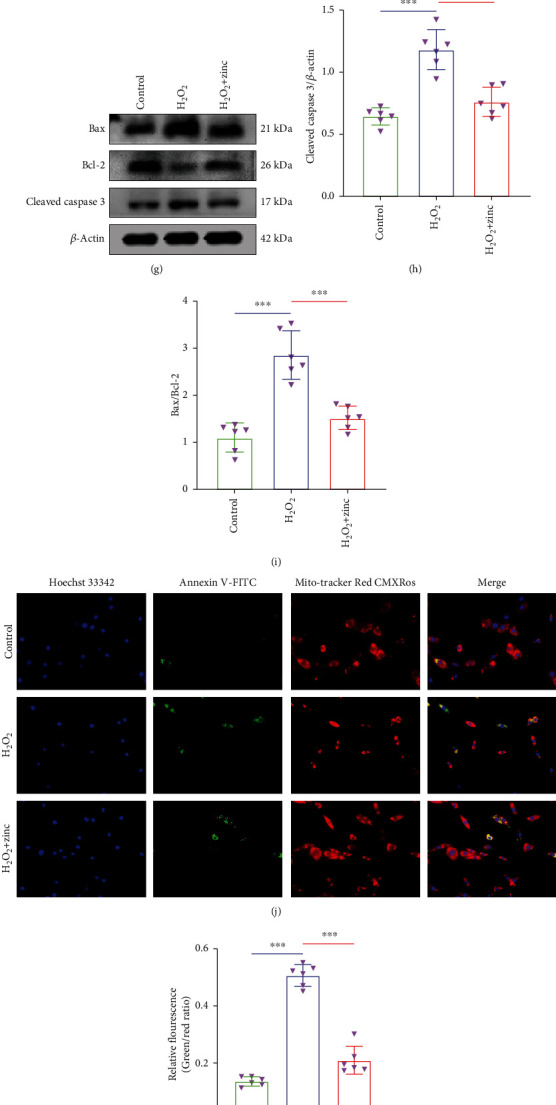
Zinc inhibited apoptosis of PC12 neurons. (a–d) The MTT results of PC12 cells stimulated by H_2_O_2_ (*n* = 6). (e) Photos of PC12 cells treated with different concentrations of H_2_O_2_ for different time. (f) Using MTT to detect the cell viability of PC12 cells after zinc treatment (*n* = 6). (g–i) The expression levels of cleaved caspase 3, Bax, and Bcl-2 proteins were detected by WB. *β*-Actin was used as internal controls for WB (*n* = 6). (j, k) Apoptosis was detected using the MMP and Apoptosis Detection Kit (*n* = 6, scale bar = 50 *μ*m). (l, m) Flow cytometry results of apoptosis (*n* = 6). Data are represented as the means ± SD. ^∗^*p* < 0.05, ^∗∗^*p* < 0.01, and ^∗∗∗^*p* < 0.001.

In order to explore the protective effect of zinc on PC12 cells, cells were first processed by H_2_O_2_. Different concentrations of H_2_O_2_ were used to stimulate PC12 cells for different time. MTT results showed that the cell viability of 60 *μ*M H_2_O_2_ for 3 hours was about 60%, which is an appropriate concentration and time. High concentration and longtime caused more cell death, which is not suitable for follow-up experiments (Figures 1(a)–1(d)). From Figure 1(e), the photos of cells, the results displayed by MTT, can be seen more intuitively. The results showed that ZnG above 100 *μ*M showed cytotoxicity to PC12, so we chose 80 *μ*M ZnG as the treatment concentration (Figure 1(f)). The specific processing method of each group of cells was shown in the method. We used WB to identify the expression levels of apoptosis key markers cleaved caspase 3, Bax, and Bcl-2. The expression levels of cleaved caspase 3 and Bax were increased in the H_2_O_2_ group comparing the control group, and the expression level of Bcl-2 was decreased, while the H_2_O_2_+zinc group reversed this result (Figures 1(g)–1(i)). The results of the Apoptosis Detection Kit showed that the green fluorescence representing apoptosis was increased in the H_2_O_2_ group, while it was decreased after zinc treatment (Figures 1(j) and 1(k)). The results of flow cytometry indicated that the percentage of apoptosis in the H_2_O_2_ group was significantly higher than that in the control group, while that in the H_2_O_2_+zinc group was lower than that in the H_2_O_2_ group (Figures 1(l) and 1(m)). These results implicated that zinc inhibited apoptosis of PC12 neurons.

### 3.2. Zinc Promoted Glucose Transport in PC12 Neurons

**Figure 2:**
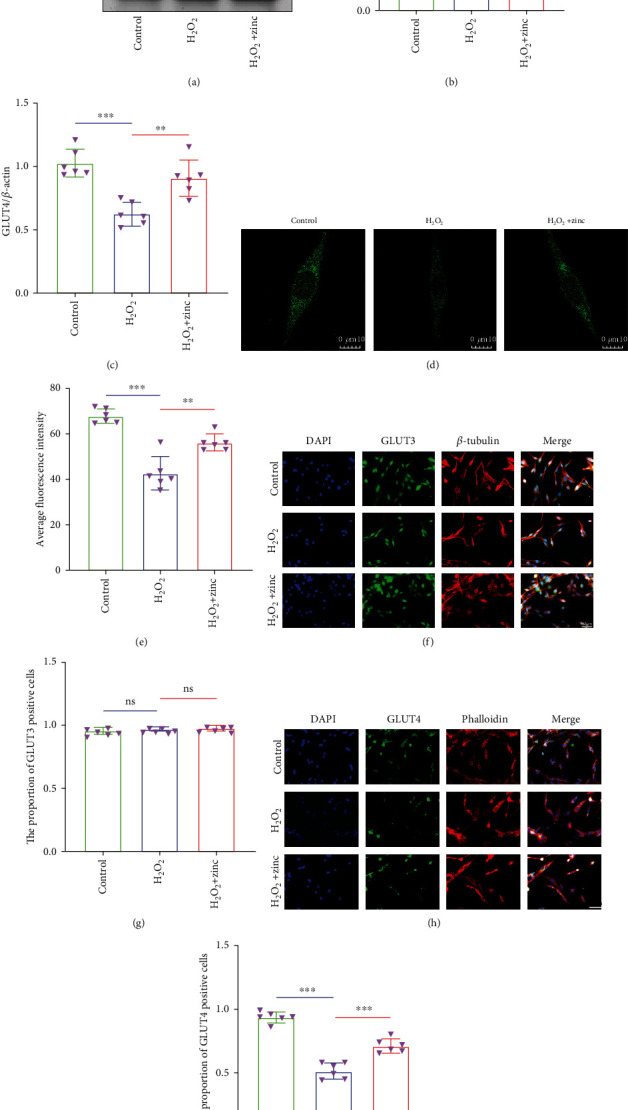
Zinc promoted glucose transport in PC12 neurons. (a–c) The expression levels of GLUT3 and GLUT4 proteins were assessed by WB (*n* = 6). (d, e) Glucose uptake assay was performed by using 2-NBDG (*n* = 6, scale bar = 10 *μ*m). (f, g) Immunofluorescence was used to detect the level of GLUT3 (cytoskeleton was labeled with *β*-tubulin) (*n* = 6, scale bar = 50 *μ*m). (h, i) The expression of GLUT4 was detected by immunofluorescence (cytoskeleton was labeled with phalloidin) (*n* = 6, scale bar = 50 *μ*m). Data are represented as the means ± SD. ^∗^*p* < 0.05, ^∗∗^*p* < 0.01, and ^∗∗∗^*p* < 0.001.

To investigate the effect of zinc on the glucose transport ability of the neurons after injury, we focused on detecting the expression level of glucose transporters. WB results suggested that the expression level of GLUT4 protein in the H_2_O_2_ group was less than that in the control group, while the level in the H_2_O_2_+zinc group was higher than that in the H_2_O_2_ group. However, there was no significant change in GLUT3 protein expression level in each group (Figures 2(a)–2(c)). Immunofluorescence also showed the same result that the proportion of GLUT4-positive cells in the H_2_O_2_+zinc group was more than that in the H_2_O_2_ group, while there was no difference in the proportion of GLUT3-positive cells (Figures 2(f)–2(i)). In addition, we used a confocal microscope to observe the consequences of cell uptake of 2-NBDG. As presented in Figures 2(d) and 2(e), the average fluorescence intensity of the H_2_O_2_+zinc group was higher than that of the H_2_O_2_ group. Collectively, these findings provided a clue that zinc promoted glucose transport in PC12 neurons after injury.

### 3.3. Zinc Had Protective Effect on Mitochondria of PC12 Neurons

**Figure 3:**
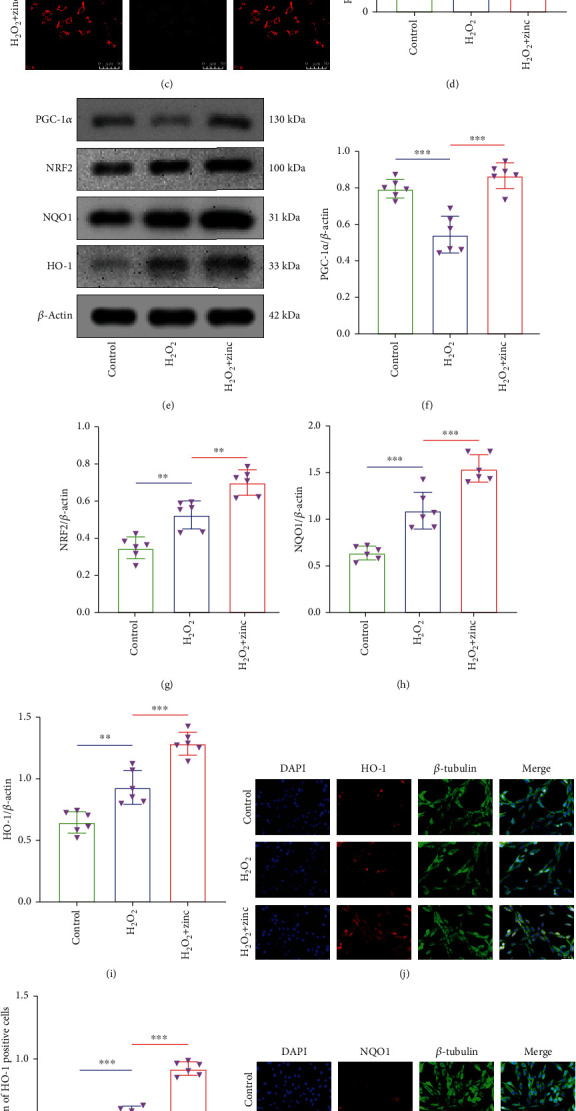
Zinc had a protective effect on mitochondria of PC12 neurons. (a, b) ROS was measured by DCFH-DA (*n* = 6, scale bar = 50 *μ*m). (c, d) Using JC-1 to detect mitochondrial membrane potential (*n* = 6, scale bar = 50 *μ*m). (e–i) The expressions of PGC-1*α*, NRF2, NQO1, and HO-1 were assessed by WB (*n* = 6). (j–m) Immunofluorescence staining was used to detect the level of NQO1 and HO-1 (*n* = 6, scale bar = 50 *μ*m). Data are represented as the means ± SD. ^∗^*p* < 0.05, ^∗∗^*p* < 0.01, and ^∗∗∗^*p* < 0.001.

We elaborated on the protective effect of zinc on PC12 neuron mitochondria from different aspects. We first used a ROS Assay Kit to measure the changes of intracellular ROS. As shown in Figures 3(a) and 3(b), the average fluorescence intensity of the H_2_O_2_ group was significantly higher than that of the control group, indicating a significant increase of intracellular ROS. After zinc treatment, intracellular ROS was decreased compared with the H_2_O_2_ group. This result reported that zinc reduced the production of mitochondrial ROS after injury. Then, the functional status of the mitochondria was detected by JC-1, which reflects the level of mitochondrial membrane potential. As it is observable from Figures 3(c) and 3(d), the red light in the H_2_O_2_ group was significantly weaker than the control group, and the green light was notably increased, reflecting that the cell mitochondrial membrane potential was markedly reduced after H_2_O_2_ stimulation. Yet, the H_2_O_2_+zinc group reversed this consequence, indicating that zinc enhanced the mitochondrial membrane potential of PC12 cells. Furthermore, mitochondrial biogenesis-related protein PGC-1*α* and oxidative stress-related proteins NRF2, NQO1, and HO-1 were detected by WB. As displayed in Figures 3(e)–3(i), the expression levels of PGC-1*α*, NRF2, NQO1, and HO-1 in the H_2_O_2_+zinc group were higher than those in the H_2_O_2_ group. These results indicated that zinc inhibited oxidative stress and promoted mitochondrial production. The immunofluorescence of NQO1 and HO-1 also illustrated the same problem (Figures 3(j)–3(m)). In summary, zinc had a protective effect on the mitochondria of PC12 neurons.

### 3.4. Zinc Regulated Glucose Metabolism in PC12 Neurons via the AMPK Pathway

**Figure 4:**
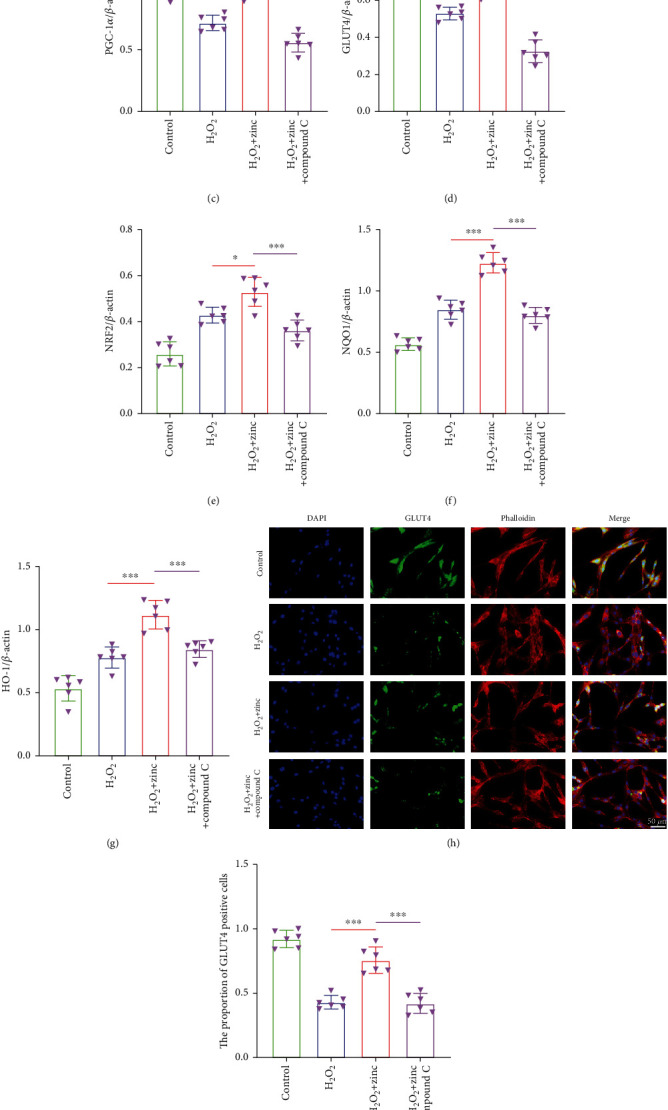
Zinc regulated glucose metabolism in PC12 neurons via the AMPK pathway. (a–g) Western blots of p-AMPK, AMPK, PGC-1*α*, GLUT4, NRF2, NQO1, and HO-1 expressions after compound C treatment (*n* = 6). (h–k) The expressions of GLUT4 and NRF2 were detected by immunofluorescence staining (*n* = 6, scale bar = 50 *μ*m). Data are represented as the means ± SD. ^∗^*p* < 0.05, ^∗∗^*p* < 0.01, and ^∗∗∗^*p* < 0.001.

To prove whether zinc regulates the glucose metabolism in PC12 neurons through the AMPK pathway, we treated the cells with the AMPK inhibitor compound C (10 *μ*M). As presented in Figures 4(a)–4(g), after zinc treatment, the expression levels of PGC-1*α*, GLUT4, NRF2, NQO1, and HO-1 proteins were increased. At the same time, the expression of p-AMPK was significantly greater than that of the H_2_O_2_ group, indicating that zinc activated the AMPK pathway. After compound C was placed on cells, p-AMPK, PGC-1*α*, GLUT4, NRF2, NQO1, and HO-1 were decreased to varying degrees, indicating that zinc worked through the AMPK pathway. Immunofluorescence staining of GLUT4 and NRF2 also proved this point, which the proportion of positive cells was notably reduced after the inhibitor was given (Figures 4(h)–4(k)). The above results indicated that zinc regulated neuronal glucose metabolism via the AMPK pathway.

### 3.5. Zinc Enhanced Glucose Transport in the Spinal Cord of SCI Mice

**Figure 5:**
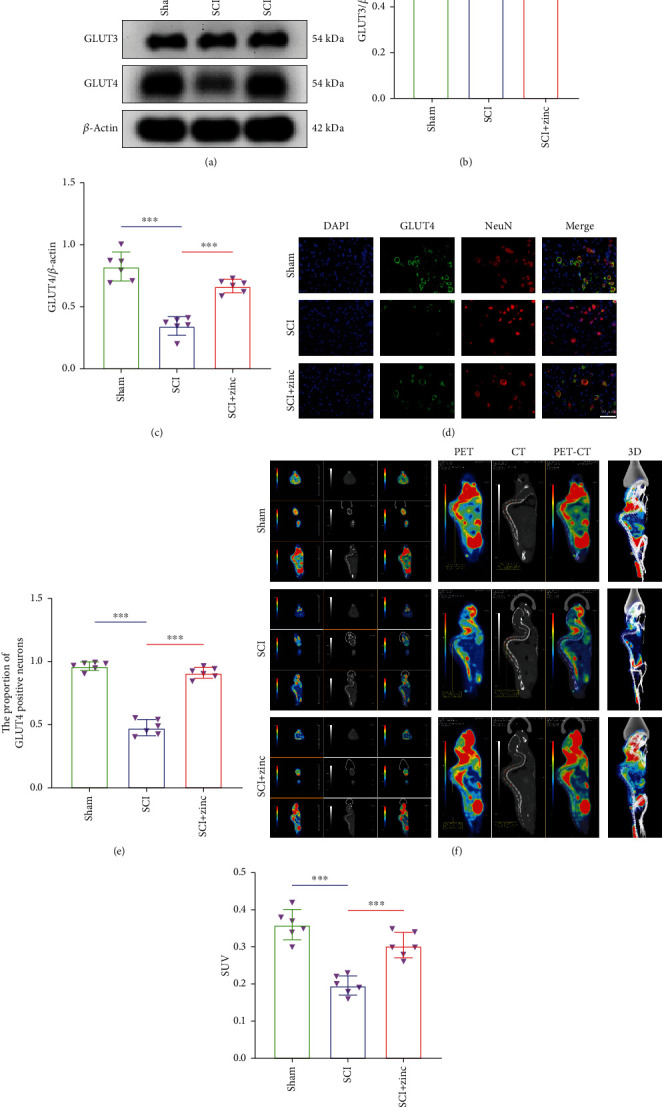
Zinc enhanced glucose transport in the spinal cord of SCI mice. (a–c) The expression levels of GLUT3 and GLUT4 proteins were assessed by WB at 3 days after injury (*n* = 6). (d, e) Immunofluorescence staining of GLUT4 at 3 days after injury (*n* = 6, scale bar = 50 *μ*m). (f, g) Glucose uptake was established by PET imaging of the biodistributions of ^18^F-FDG at 3 days after injury (*n* = 6). Data are represented as the means ± SD. ^∗^*p* < 0.05, ^∗∗^*p* < 0.01, and ^∗∗∗^*p* < 0.001.

Three days after treated with zinc, SCI mice were detected by WB, immunofluorescence staining, and PET-CT. Consistent with the cell experiment results, the WB results showed that the expression of GLUT4 protein in the SCI group was lower than that in the sham group, while the SCI+zinc group was higher than that in the SCI group. There was little change in the expression of GLUT3 among the three groups (Figures 5(a)–5(c)). Immunofluorescence staining of GLUT4 suggested that the proportion of GLUT4-positive neurons in spinal anterior horn in the SCI group was reduced, but it was close to the normal level after zinc treatment (Figures 5(d) and 5(e)). In order to study the changes in glucose uptake further, we used PET-CT to detect the distribution of ^18^F-FDG in mice. As displayed in Figures 5(f) and 5(g), the average standardized uptake value (SUV) of ROI of the SCI group was lower than that of the sham group, while it was improved compared with the SCI group after zinc administration. The above data revealed that zinc enhanced glucose transport in SCI mice.

### 3.6. Zinc Protected the Spinal Cord Mitochondria in SCI Mice

**Figure 6:**
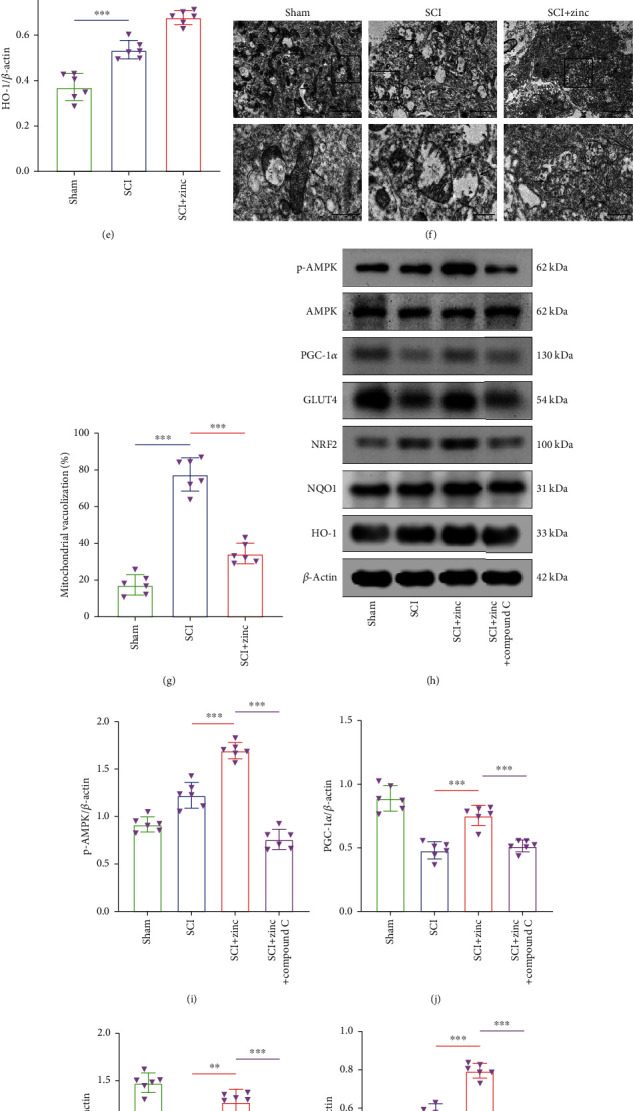
Zinc regulated glucose metabolism in the spinal cord of SCI mice through the AMPK pathway. (a–e) The expression levels of PGC-1*α*, NRF2, NQO1, and HO-1 proteins were assessed by WB at 3 days after injury (*n* = 6). (f, g) Transmission electron microscopy was used to monitor mitochondrial injury in the spinal cord (*n* = 6, scale bar = 2 *μ*m and 500 nm). (h–n) Western blots of p-AMPK, AMPK, PGC-1*α*, GLUT4, NRF2, NQO1, and HO-1 expression after compound C treatment at 3 days after injury (*n* = 6). Data are represented as the means ± SD. ^∗^*p* < 0.05, ^∗∗^*p* < 0.01, and ^∗∗∗^*p* < 0.001.

WB and TEM were used to prove the protective effect of zinc on the mitochondria of SCI mice. Results of WB suggested that zinc enhanced the expression levels of PGC-1*α*, NRF2, NQO1, and HO-1 protein compared with the SCI group (Figures 6(a)–6(e)). Next, we used TEM to observe the morphological changes of the spinal cord mitochondria. As presented in Figures 6(f) and 6(g), it could be seen that there were more mitochondria with normal morphology and abundant mitochondrial cristae in the sham group. While in the SCI group, the number of mitochondria was reduced, the mitochondrial cristae were disappeared, and the mitochondrial vacuolization was significantly increased. After zinc treatment, the mitochondria morphology has been repaired, and the vacuolization was reduced. Those results indicated that zinc reduced oxidative stress, promoted mitochondrial production, and had a protective effect on the spinal cord mitochondria in SCI mice.

### 3.7. Zinc Regulated Glucose Metabolism in the Spinal Cord of SCI Mice through the AMPK Pathway

In order to clarify the mechanism of action of zinc, like cells, we injected compound C, an AMPK inhibitor, into the abdominal cavity of mice. The specific dosage was shown in Materials and Methods. We used WB to detect the expression levels of p-AMPK, AMPK, PGC-1*α*, GLUT4, NRF2, NQO1, and HO-1 proteins. The results of Figures 6(h)–6(n) showed that zinc increased the expression of p-AMPK, PGC-1*α*, GLUT4, NRF2, NQO1, and HO-1 compared with the SCI group, while the SCI+zinc+compound C group decreased the expression of these proteins compared with the SCI+zinc group, indicating that zinc regulated glucose metabolism in the spinal cord of SCI mice through the AMPK pathway.

### 3.8. Zinc Promoted the Recovery of Motor Function of SCI Mice via the AMPK Pathway

**Figure 7:**
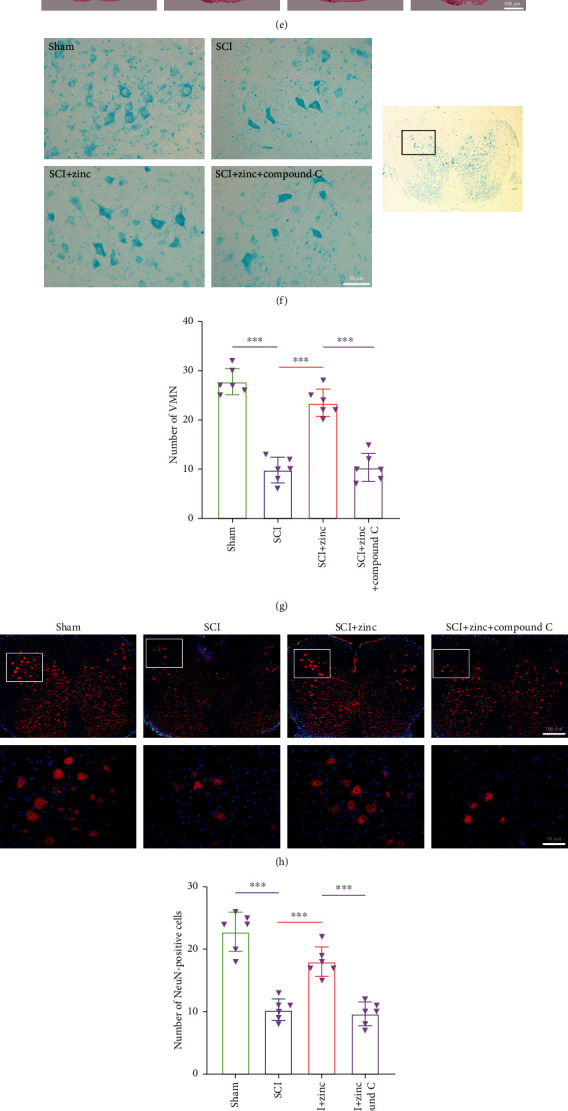
Zinc promoted the recovery of motor function of SCI mice via the AMPK pathway. (a) BMS scores were performed to evaluate the motor function recovery at 1, 3, 5, 7, 10, 14, and 28 days after the operation (*n* = 12). (b–d) Footprint analysis was performed at 28 days after injury. Stride length and width were counted (*n* = 6). (e) The HE staining at 28 days post SCI (scale bar = 50 *μ*m and 300 *μ*m). (f, g) Nissl staining was used to assess the loss of the neurons at 28 days, and the number of ventral motor neuron (VMN) was counted (*n* = 6, scale bar = 50 *μ*m). (h, i) Immunofluorescence staining of NeuN at 28 days post SCI (*n* = 6, scale bar = 50 *μ*m and 200 *μ*m). (j, k) TUNEL staining at 3 days post SCI (*n* = 6, scale bar = 50 *μ*m). Data are represented as the means ± SD. ^∗^*p* < 0.05, ^∗∗^*p* < 0.01, and ^∗∗∗^*p* < 0.001.

To investigate the effects of zinc on the recovery of function, we first performed the BMS score and footprint analysis. As shown in Figure 7(a), the BMS score of the SCI+zinc group was higher than that of the SCI group at the 14th day, and there was a significant difference between two groups at the 21st day and the 28th day. BMS score of mice after compound C treatment was lower than that in the SCI+zinc group. As displayed in Figures 7(b)–7(d), the sham group mice walked in a straight line with gait coordination. After SCI, the hind limbs of mice could not stand, and the gait was obviously uncoordinated. After zinc treatment, the hind foot of mice could touch the ground, and the gait was more stable than that of the SCI group. After compound C was given, the hind limbs of the mice could only stand occasionally. At the same time, stride length and stride width of the mice were improved after zinc treatment, and compound C reversed the effect of zinc. In order to explain the changes in motor function further, a morphological test was carried out. As shown by HE, the spinal cord in the SCI group was flattened and fragmented, with more cavities, and a large number of the neurons were damaged. Compared with the SCI group, the spinal cord in the SCI+zinc group was markedly improved with the neurons slightly damaged. After compound C treatment, the effect of zinc was reversed (Figure 7(e)). In order to describe the damage and recovery of the neurons more objectively, we implemented Nissl staining and neuron-specific immunofluorescence staining. The results of Nissl staining indicated that the number of ventral motor neuron (VMN) after SCI was significantly reduced compared with the sham group. After zinc treatment, the number was increased compared with the SCI group, while after the inhibitor was administered, it was lower than that of the SCI+zinc group (Figures 7(f) and 7(g)). As displayed in Figures 7(h) and 7(i), the number of NeuN-positive cells in spinal anterior horn in the SCI group was reduced compared with that in the sham group. After zinc treatment, the number of cells was significantly higher than that of the SCI group. After adding compound C, the number of NeuN-positive cells was lower than that of the SCI+zinc group. In addition, we performed TUNEL staining. The results showed that the rate of TUNEL-positive cells in the SCI group was notably higher than that in the sham group, and it was decreased after zinc administration. The incidence in the SCI+zinc+compound C group was higher than that in the SCI+zinc group (Figures 7(j) and 7(k)). The above results suggested that zinc promoted the recovery of motor function and protected neurons in SCI mice through the AMPK pathway.

## 4. Discussion

**Figure 8:**
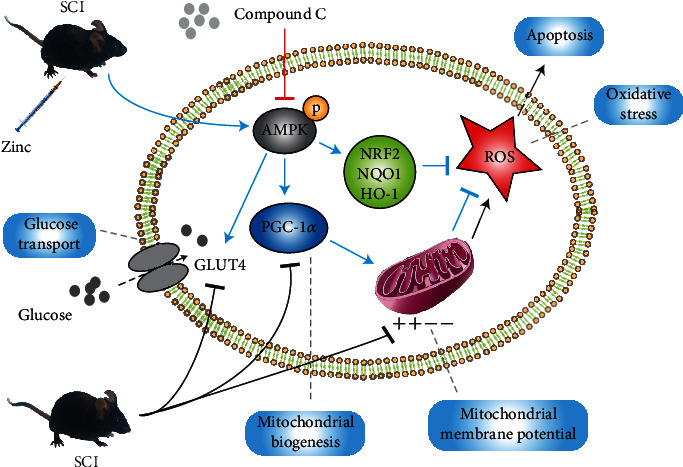
The scheme of zinc regulating neuronal glucose metabolism after SCI. After SCI, neuronal glucose transport, mitochondrial biogenesis, and mitochondrial membrane potential of the neurons were decreased, with ROS accumulating and oxidative stress increasing (black arrow). Through the AMPK pathway, zinc strengthened neuronal glucose transport, promoted mitochondrial biogenesis, increased mitochondrial membrane potential, and reduced oxidative stress after SCI (blue arrow). Compound C suppressed the effect of zinc (red arrow).

In the present study, we determined the regulatory effect of zinc on the spinal cord and neuronal glucose metabolism after SCI. We first constructed a cellular oxidative stress model and a mouse SCI model to estimate the effects of zinc in vitro and in vivo. Glucose transport and utilization of glucose (protection of mitochondria) are essential for glucose metabolism, so this experiment started with research on two aspects of glucose uptake and protection of mitochondria. At the same time, the role of AMPK was tested and the mouse motor function was evaluated. Our results suggested that zinc enhanced the glucose uptake of the injured neurons and spinal cord, reduced oxidative stress and neuronal apoptosis, promoted mitochondrial biogenesis, and encouraged recovery of motor function of SCI mice. After the administration of AMPK inhibitor, all the effects of zinc had changed, which proved that the regulation of zinc on neuron and spinal cord glucose metabolism after SCI was mainly achieved through the AMPK signaling pathway (Figure 8).

First, we tested the effect of zinc on glucose transport after SCI. Glucose is the main energy supplier of the central nervous system, and the utilization of glucose ensures a large amount of energy required for neuronal activity [30]. In a variety of diseases related to the nervous system, the disorder of glucose metabolism is an important step in the occurrence and progression of the disease [31]. Disturbance of glucose metabolism caused by spinal cord ischemia and hypoxia is an important part of secondary damage of SCI. The transmembrane ion balance of the neurons requires continuous glucose energy supply. Studies have shown that energy fluctuations caused by changes in glucose uptake are closely linked to the survival of the neurons [5, 9]. After SCI, the glucose uptake of the spinal cord of rodents was decreased, with the metabolism disturbance, and neuronal activity was inhibited [32]. The entry of glucose into cells depends on glucose transporters. There are subtypes of glucose transporters, among which the most abundant expressions in the neurons are GLUT3 and GLUT4 [6]. Damage can cause GLUT dysfunction, which in turn leads to impaired glucose uptake and metabolism [8]. In vitro, we measured the expression of glucose transporters by WB and immunofluorescence, and assessed the glucose uptake of PC12 cells by 2-NBDG, which proved that zinc promoted glucose transport in the neurons after injury. In vivo, we first detected the expression of glucose transporters, and then visually assessed spinal cord glucose uptake by PET-CT, concluding that zinc enhanced spinal cord glucose transport after SCI. In short, zinc promoted the glucose uptake of the spinal cord and neurons after SCI, which was conducive to the recovery of nerve function.

Then, we identified the protective effect of zinc on mitochondrial function after SCI. Mitochondria are organelles with a double-layer membrane structure in eukaryotes [10]. They are significant regulators of cellular energy homeostasis and are particularly important for cell life [9]. There are numerous differences in the number, size, and shape of mitochondria in different cells. Neural cells that require a lot of energy contain about 1000-2000 mitochondria [10]. Mitochondria are involved in cellular glucose metabolism, amino acid production, lipid metabolism, ion homeostasis, and oxidation regulation [33]. In the secondary injury of SCI, mitochondrial dysfunction is a link that cannot be ignored, which can cause energy disorders, ROS accumulation, and neuronal death [30]. The repair of the neurons after injury, including axon regeneration, is inseparable from the supply of energy from well-functioning mitochondria [1]. The peroxisome proliferator-activated receptor (PPAR) *γ* coactivator *α* (PGC-1*α*) is an important regulator of mitochondrial function, especially playing a key role in mitochondrial biogenesis [34]. Previous studies have revealed that the expression of PGC-1*α* was down in a rat model of SCI [30]. In this experiment, we proved that zinc elevated the level of PGC-1*α* in the neurons and spinal cord of mice after injury, which is conducive to mitochondrial production. Oxidative stress involves a series of neurological diseases and is a serious secondary injury after SCI [1]. Mitochondria are both the main source of ROS and the main victim of ROS [10]. With the progress of oxidative stress and the accumulation of ROS, the function of mitochondria is further destroyed, which is not conducive to the recovery of nerve function. Therefore, in the process of protecting mitochondria, antioxidant therapy is indispensable. Nuclear factor E2-related factor 2 (NRF2) is recognized as a key factor in regulating oxidative stress and is associated with the regulation of mitochondrial function [12]. NRF2 regulates the expression of phase II detoxification enzymes NADH dehydrogenase quinone 1 (NQO1) and heme oxygenase-1 (HO-1). The latter as antioxidant enzymes play an important role in oxidative stress and increasing cellular antioxidant capacity [35]. In this experiment, we found that zinc enhanced the expression of oxidative stress-related proteins NRF2, NQO1, and HO-1, and played an antioxidant effect. We detected changes in intracellular ROS and mitochondrial membrane potential, and proved that zinc reduced the generation of neuronal ROS while enhancing mitochondrial function. The morphology of the mitochondria was observed by transmission electron microscope, and it was found that zinc effectively decreased mitochondrial vacuolization after SCI. In summary, zinc had a protective effect on mitochondria after injury.

Next, we clarified the impact of the AMPK pathway in the role of zinc. AMPK is a serine/threonine kinase, an energy sensor for cells, and a key molecule in glucose metabolism [36]. The AMPK signaling pathway is closely related to mitochondrial biogenesis, energy supply, and oxidative stress [12]. Studies have shown that the phosphorylation of AMPK enhanced the expression of GLUT4 in cells, which was essential for glucose uptake [11]. Our previous study has proved that zinc had an activating effect on AMPK [37]. In this experiment, we used AMPK inhibitor compound C to verify the influence of the AMPK pathway on the role of zinc. The results showed that the expressions of PGC-1*α*, GLUT4, and oxidative stress-related proteins, enhanced by zinc, were all effectively inhibited by compound C. At the same time, the protection of zinc on neuronal apoptosis and the promotion of the recovery of mouse motor function were all weakened to varying degrees by the addition of compound C. In short, zinc's effects of regulation of glucose metabolism and recovery of exercise function were achieved to some extent through the AMPK pathway.

It is worth pointing out that this experiment has certain limitations. First of all, this experiment used the PC12 cell line as the research object. Although it is a neuronal cell line, it cannot replace the neurons and primary neurons should be used to show more convincing data. Secondly, the study on the mechanism of zinc regulating glucose metabolism in this experiment is not thorough, and the connection between the mitochondria and glucose transport has yet to be elucidated. Finally, we have not studied the products of glucose metabolism. Our team plans to use high-throughput methods such as transcriptomics and metabolomics to study the in-depth regulation of zinc on glucose metabolism in the spinal cord of mice after SCI.

## 5. Conclusion

Our current research has demonstrated that zinc regulated the glucose metabolism of the spinal cord and neurons after SCI partially through the AMPK pathway. Our results suggested that zinc promoted spinal cord glucose transport, protected the mitochondrial function, reduced neuronal apoptosis, and improved motor function recovery after SCI. Zinc therapy is expected to become a potential strategy for the treatment of SCI.

## Data Availability

The raw data of experiments used to support the findings of this study are available from the corresponding authors upon request.
